# Contribution of ammonia-oxidizing archaea and bacteria to nitrogen transformation in a soil fertilized with urea and organic amendments

**DOI:** 10.1038/s41598-023-44147-x

**Published:** 2023-11-25

**Authors:** Yajun Yang, Hexiang Liu, Yang Zhang, Xianhui Fang, Xianbao Zhong, Jialong Lv

**Affiliations:** 1 College of Natural Resources and Environment, Northwest A&F University, Yangling, 712100 Shaanxi People’s Republic of China; 2 Key Laboratory of Plant Nutrition and the Agri-Environment in Northwest China, Ministry of Agriculture, Yangling, China

**Keywords:** DNA, Environmental chemistry, Geochemistry

## Abstract

The contribution of ammonia-oxidizing archaea (AOA) and ammonia-oxidizing bacteria (AOB) is crucial for nitrogen transformation. The effects of four organic amendments (OAs) plus urea on soil nitrogen transformation and the contribution of the ammonia-oxidizing microbial community were investigated using an incubation experiment. The OAs plus urea treatments included pig manure plus urea (PM + U), wheat straw plus urea (WS + U), compost plus urea (CP + U) and improved-compost plus urea (IC + U), while no OAs and urea amended control was noted as CK. The abundance and composition of AOA and AOB were determined using high through-put sequencing. Compared with CK, the OA plus urea treatments significantly enhanced the amount of total mineralized nitrogen released during the incubation process. After incubation, the highest mineralized nitrogen and net nitrogen mineralization was under the PM + U treatment and the lowest was in the WS + U treatment. In conclusion, among all OA plus urea treatments, the microbial biomass nitrogen content was the highest in WS + U treatment and dissolved organic nitrogen content was the highest with the PM + U treatment. Additionally, the abundance of AOB was inhibited in comparison to that of AOA; however, AOB contributed more to nitrification than AOA. Soil NO_3_^−^-N and dissolved organic nitrogen were the principal components influencing the distribution of AOA and AOB. The result illustrated that the OAs plus urea, especially PM plus urea promoted mineralization to produce more dissolved organic nitrogen and NH4^+^-N, thus accelerating the growth of AOB to strengthen nitrification in soil.

## Introduction

In recent years, long-term or excessive nitrogen applications have resulted in lower nitrogen use efficiency, lower soil quality and inorganic nitrogen leaching^1^. To promote sustainable eco-agriculture, the application of different organic amendments (OAs) based on reducing inorganic fertilizers has been widely discussed and used. These OAs contain abundant nutrients, which can be slowly released to meet the nutritional needs of crop growth and maintain organic matter, and reduce the amount of fertilizer applications in agricultural systems^2^.

Soil organic matter contains many nutrients, which can improve soil physical, chemical and biological properties. These organic substances containing organic nitrogen can provide effective nutrients for plants through decomposition and mineralization^3^. The extent of nitrogen mineralization determines the content of inorganic nitrogen in plants.

Nitrogen mineralization is a complex biological process. Mineralization can provide ammonium nitrogen for nitrification. Nitrification is an important step in nitrogen cycling in soil. Nitrification generally refers to the process by which ammonia or ammonium salts are oxidized to nitrates and nitrites by microorganisms under aerobic conditions^4^. Ammonia-oxidizing archaea (AOA) and ammonia-oxidizing bacteria (AOB) are the main nitrifiers during ammonia oxidation, which is the key process of nitrification^5^. The distribution^6,7^, abundance^8^, community composition^9^ and influencing factors of AOA and AOB have been researched extensively. In addition, the amoA gene which catalyzes ammonia oxidation, can be applied as a biomarker for AOA and AOB^10,11^.

Substantial research has found that AOA role is higher than that of AOB, both quantitatively and functionally, in many soils^12,13^. Usually, the quantitative advantage of ammonia oxidizers is related to their nitrification function. However, some studies have shown that AOA dominated nitrification in quantity and AOB dominates in function^14,15^. Lennon et al. have shown that in soil, many microorganisms are present in large numbers, but they do not contribute actively to the function of the ecosystem^16^. Therefore, the abundances of AOA and AOB, as well as their contributions to nitrification in soil, will vary under different circumstances.

The amounts of nitrogen mineralization and nitrification depend on the quantity and composition of organic matter, microbial activity and various environmental conditions^17,18^. Much research has been conducted on the nitrogen mineralization dynamics of OAs such as animal manure, compost, and straw, in agricultural systems^19,20^. However, few studies have compared the effects of different OAs plus urea on nitrogen mineralization and nitrification dynamics as well as the changes in ammonia oxidizers. Therefore, this study focused on the impact of four kinds of OAs plus urea, pig manure plus urea (PM + U), wheat straw plus urea (WS + U), compost plus urea (CP + U), and improved-compost plus urea (IC + U), on soil nitrogen mineralization and nitrification as well as the dynamics of AOA and AOB. The aims of this study included (1) investigate how soil nitrogen mineralization, nitrification, and abundance and diversity of AOA and AOB react to OAs plus urea and (2) explore the relative contribution of AOA and AOB communities to soil nitrification with OA amendments plus urea.

## Materials and methods

### Soil and materials tested

The soil was obtained using the soil-drilling method on June 25, 2019 from the top layer (0–20 cm) of a farmland (34° 42′ N and 108° 09′ E, 1050 m above sea level) that had not been planted for many years. The tested soil was classified as yellow-cinnamon based on soil taxonomy. The tested soil had 11.5% (w/w) natural field moisture, and a portion of the soil samples were air-dried at room temperature for 2 months before use for chemical determination. Residual roots, litter and stones were removed from the field-moist soil by passing through a 2-mm sieve and then stored at – 20 °C for incubation in the laboratory. The soil had a pH of 7.53, total C content of 7.90 g kg^−1^, total N content of 0.75 g kg^−1^, Olsen phosphorus of 17.5 mg kg^−1^, available potassium of 172.0 mg kg^−1^, bulk density of 1.2 g cm^−3^, silty content (0.02–0.002 mm) of 61.56%, clay content (< 0.002 mm) of 6.60% and sand content (2–0.02 mm) of 31.84%.

PM and WS were collected from commercial piggery and local farmland, respectively. CP was obtained by a 56-day aerobic compost using PM and WS at a proportion of 1:2 (on a dry weight basis). IC samples was prepared under co-composting with WS, PM, 10% biochar, and 15% bean dregs (based on dry weight of PM). Biochar, purchased from Yangling Pvt. Lvd., Shaanxi province, was obtained from wheat straw through slow and dry pyrolysis at 500–600 °C. The amendments were chopped using a crusher and passed through a 2 mm sieve for testing. The chemical properties of the OAs used in the 77-day incubation experiment were determined as per the protocols described by TMECC^21^ and are presented in Table 1. The highest carbon concentration and lowest nitrogen content was in WS among all OAs. The lowest total nitrogen content of WS resulted in the highest C/N ratio. The C/N ratios of the four amendments from the lowest to highest were as follows: PM < CP < IC < WS.

**Table 1:** Chemical properties of organic amendments used in 97-day incubation experiment.

Parameter	PM	WS	CP	IC
C (%DM)	31.55 (0.14)	40.66 (0.12)	27.25 (0.09)	32.19 (0.05)
N (%DM)	2.44 (0.02)	1.09 (0.03)	2.09 (0.01)	2.45 (0.33)
C/N ratio	12.93	37.34	13.03	13.32
pH	6.56 (0.06)	8.21 (0.01)	7.79 (0.08)	8.15 (0.07)
Total P (%DM)	1.81 (0.11)	0.18 (0.02)	0.92 (0.48)	1.28 (0.20)
Total K (%DM)	1.17 (0.08)	0.12 (0.01)	3.82 (0.23)	3.35 (0.35)

*n.d* not determined, *C* carbon, *N* nitrogen, *P* phosphorus, *K* potassium, *DM* dry matter, *PM* pig manure, *WS* wheat straw, *CP* compost, *IC* improved compost.

### Experimental design

Four soil-amended treatments, pig manure plus urea (PM + U), wheat straw plus urea (WS + U), compost plus urea (CP + U), improved compost plus urea (IC + U) and a control (no urea and organic amendments, CK) with three replicates were carried out at 25 °C in the 77-day laboratory incubation experiment. The amount of total nitrogen application was 0.5 mg g^−1^ (dry soil, N dose was 10 times that of the field application) in all soils. Nitrogen from urea (70%) and that obtained from OAs (30%) was applied based on their nitrogen content in PM + U, WS + U, CP + U and IC + U treatments. After calculations, a total of 9.63 g, 4.30 g, 5.02 g, and 4.29 g of WS, PM, CP, and IC samples (dry weight), respectively, was mixed uniformly with 700 g of air-dried soil and then placed in a 1.2 L plastic box for each treatment. A plastic film with small holes was used to cover the boxes for air circulation. All treatments were maintained field water holding capacity at 70% during the incubation period, and the weight loss in each box was replaced every 3 days with distilled water. At each sampling point, 0, 3, 7, 14, 21, 49 and 77 days of incubation, approximately 100 g of samples were collected from the entire depth in each plastic box by destructive sampling.

### Determination methods

Soil organic carbon was determined using the K_2_Cr_2_O_7_ digestion method. Total nitrogen (TN) was measured using the Kjeldahl digestion method. Soil pH was measured using a pH meter with a soil-to-water ratio of 1:2.5 (w/v). Olsen phosphorus was extracted using 0.5 mol L^−1^ NaHCO_3_ and determined using the molybdenum blue method^2^. Soil available potassium was determined by flame photometry (6400A, INESA, China) after extraction with 1 mol L^−1^ NH_4_OAc^22^. The soil bulk density was determined using the cutting ring method.

The microbial biomass nitrogen (MBN) was determined by chloroform fumigation-K_2_SO_4_ extraction method. A sample of 5 g soil was drawn with 40 mL of 1 mol L^−1^ KCL with swing for 2 h, leached through a 0.45 microporous membrane after centrifugation for 10 min, and finally, the dissolved organic nitrogen (DON) was measured using a total organic carbon analyzer (Wu et al. 2017). In addition, the mineral nitrogen (MN; NH_4_^+^-N and NO_3_^−^-N) was measured using a continuous flow autoanalyzer (AA3). The net N mineralization was calculated as per Mohanty et al*.*^18^. The percentage of MN to total applied N was calculated as follows^23^:

$$\% {\text{mineralization}} = \frac{MN(amended) - MN(control)}{{NTotal(applied)}} \times 100$$

### Microbial community analysis

The samples collected from soil were stored at − 20 °C to analyze the microbial community. The structure of the ammonia oxidizer gene communities was determined using high through-put sequencing. The PCR was amplified with the following primers: Arch-*amo*AF/Arch-*amo*AR and *amo*A-1F/*amo*A-2R^11,24^ for AOA and AOB, respectively. The PCR products were verified using 1.5% agarose gel electrophoresis, and DNA concentration and quality were determined after excising and purification. Equal quantities of DNA samples were mixed and sequenced using the Illumina Miseq PE300 platform (Illumina, Inc., CA, USA) by Beijing Auwigene Tech, Ltd. (Beijing, China). After obtaining the high quality sequences, operational taxonomy units (OTUs) were clustered using a 100% similarity threshold and classified according to taxonomic identity (phylum, family, and genus level) using the Ribosomal Database Project. Chao 1 and Shannon indices were applied to indicate the richness of AOA and AOB, respectively. The AOA and AOB abundances were quantitatively measured in triplicate using qPCR. The primers for each gene in the compost samples were the same as those listed above.

### Statistical analyses

All parameters (selected properties of soils and OAs, different forms of nitrogen, and abundance and composition of the bacterial community) were measured for three replicates. The analyses were performed using Origin 2016. Correlation analysis was carried out in the SPSS v.16.0, and significant differences between treatments were compared using the least significant difference test at *P* < 0.05. Redundancy analyses (RDA) of environmental factors and ammonia oxidizer genes (AOA and AOB) were performed using Canoco 5.0.

## Results

### Changes in NH_4_^+^-N and NO_3_^—^N

**Figure 1:**
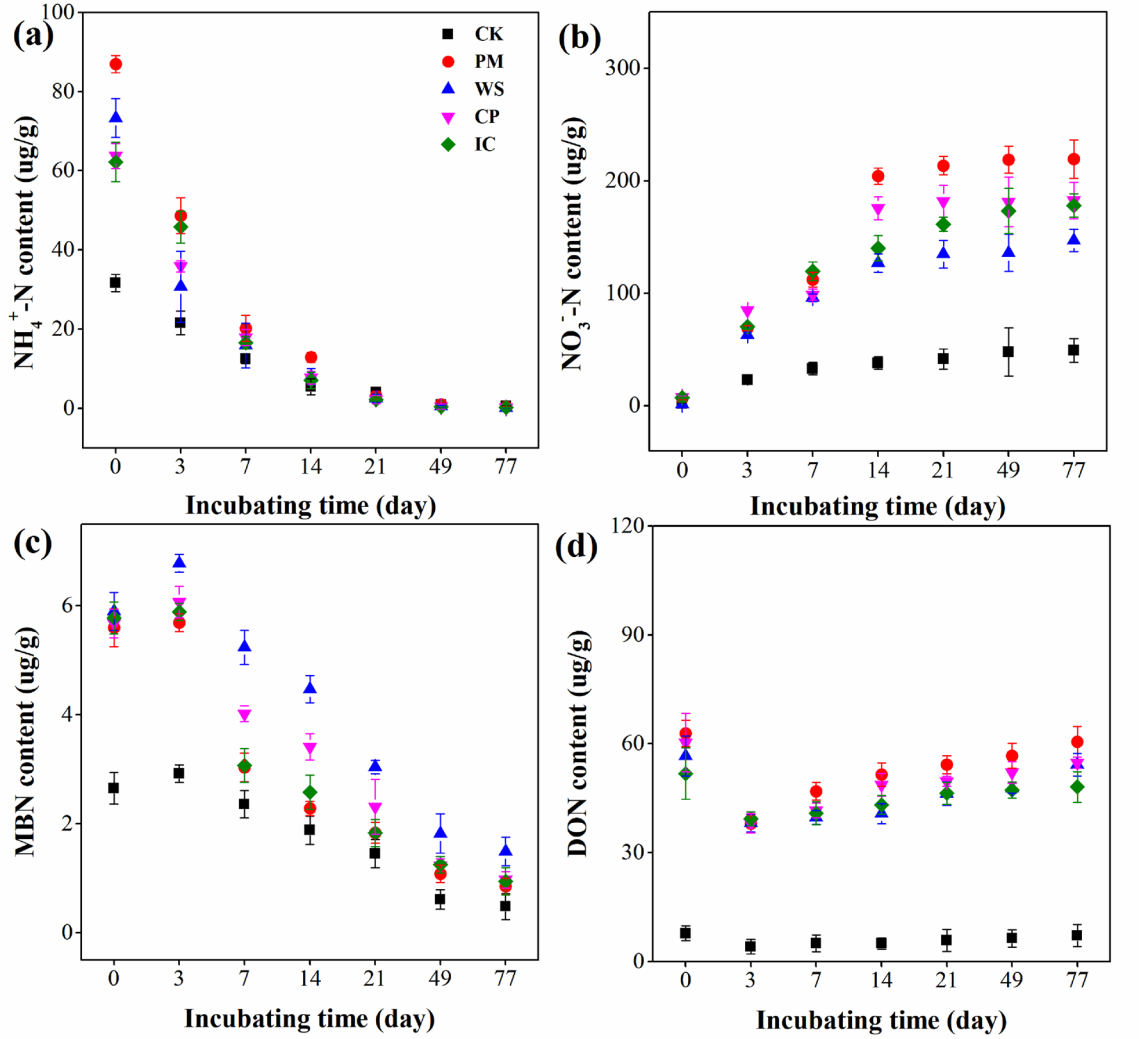
Changes in NH_4_^+^-N (**a**), NO_3_^−^-N (**b**), MBN (**c**) and DON (**d**) among all treatments during the incubation (*MBN* microbial biomass nitrogen, *DON* dissolved organic nitrogen, *PM + U* pig manure plus urea, *WS + U* wheat straw plus urea, *CP + U* compost plus urea, *IC + U* improved compost plus urea).

The NH_4_^+^-N concentration varied among all treatments, as shown in Fig. 1a. The peak value of NH_4_^+^-N was observed during the initial phase of incubation. The maximum NH_4_^+^-N content was in the PM + U treatment (86.93 μg g^−1^), followed by that in WS + U (73.30 μg g^−1^), CP + U (63.77 μg g^−1^), IC + U (62.21 μg g^−1^) and CK (31.68 μg g^−1^) treatments. Then, the NH_4_^+^-N content decreased sharply within 21 days in all treatments. The rate of decrease in NH_4_^+^-N content was within 96.33–96.53% in the OA plus urea treatments, but it was only 87.31% in the CK. Additionally, the minimum NH_4_^+^-N content was observed at the end of incubation.

The NO_3_^−^-N concentration dynamics for all treatments are shown in Fig. 1b. The NO_3_^−^-N values (1.64–7.92 μg g^−1^) were at a minimum in OA plus urea and CK treatments at the start of incubation. The NO_3_^−^-N concentration increased sharply until day 14 and then increased slowly to the maximum value until the end. The highest final content of NO_3_^−^-N was under the PM + U treatment (219.4 μg g^−1^), followed by that in CP + U (182.6 μg g^−1^), IC + U (178.1 μg g^−1^), WS + U (147.1 μg g^−1^), and CK (49.35 μg g^−1^) treatments, respectively.

### Changes in MN and net N mineralization

The MN content of all OA plus urea treatments and CK increased during the incubation process. The MN ranged within 69.52–219.8 μg N g^−1^ soil in OA plus urea treatments, whereas it was 33.81–49.93 μg N g^−1^ soil in the CK (Table 2). Total MN released in the PM + U treatment increased from 93.58 μg N g^−1^ soil to 219.8 μg N g^−1^ soil. During the entire incubation phase, the highest total MN was released in PM + U treatment (219.8 μg N g^−1^ soil) and the lowest was under the CK treatment (49.93 μg N g^−1^ soil). Additionally, the percentage of MN was the highest with the PM + U treatment and lowest with the WS + U treatment among all OA plus urea treatment groups. Compared with CK, the OA plus urea treatments significantly enhanced the MN content (*P* < 0.05). No significant difference in MN was observed between the CP + U and IC + U treatments.

**Table 2:** Total mineral nitrogen (MN) and net total mineral nitrogen (net MN) released (μg N g^−1^ soil) from treatments with amendment and control during a 77-day laboratory incubation experiment.

Time (days)	Treatment									
	CK		PM + U		WS + U		CP + U		IC + U	
	MN	Net MN	MN	Net MN	MN	Net MN	MN	Net MN	MN	Net MN
0	33.81	–	93.58	59.77	74.94	41.13	71.69	37.88	69.52	35.71
3	44.65	–	117.8	73.17	94.02	49.37	120.9	76.25	116.4	71.70
7	45.76	–	132.4	86.60	112.1	66.39	116.8	71.03	136.3	90.52
14	43.63	–	217.1	173.5	135.1	91.48	183.5	139.9	147.3	103.7
21	45.69	–	216.7	171.0	137.6	91.87	184.2	138.5	163.7	118.0
49	48.93	–	219.9	170.9	136.5	87.59	181.7	132.8	173.8	124.8
77	49.93	–	219.8	169.8	147.2	97.30	182.8	132.8	178.4	128.4

*PM + U* pig manure plus urea, *WS + U* wheat straw plus urea, *CP + U* compost plus urea, *IC + U* improved compost plus urea.

The net N mineralization is the difference in MN between OA plus urea and CK treatments (Table 2). The net N mineralization increased from day 0 to day 14 in all soils. The maximum MN was observed in the PM + U treatment (173.5 μg N g^−1^ soil) at day 14. Afterwards, net N mineralization decreased in PM + U (from 173.5 to 169.8 μg N g^−1^ soil), WS + U (from 91.87 to 87.59 μg N g^−1^ soil), and CP + U (from 139.9 to 132.8 μg N g^−1^ soil) treatments, but increased in IC + U treatment (from 103.7 to 128.4 μg N g^−1^ soil). Net N mineralization was the highest in the PM + U group and lowest in the WS + U group among all OA plus urea added soils.

### Changes in MBN and DON with different OAs plus urea

The change in MBN content was similar in all treatments during the incubation period (Fig. 1c). The MBN content increased to the maximum value on day 3, and declined gradually thereafter in all treatments. The maximum MBN values reached were 5.69 μg g^−1^, 5.89 μg g^−1^, 6.07 μg g^−1^, and 6.78 μg g^−1^ in the PM + U, IC + U, CP + U, and WS + U groups, respectively, whereas the lowest MBN content was in the CK (2.92 μg g^−1^). At the end of incubation, the MBN value was 0.85 μg g^−1^, 0.94 μg g^−1^, 0.97 μg g^−1^, and 1.49 μg g^−1^ under the PM + U, IC + U, CP + U, and WS + U treatments, respectively, while the lowest MBN concentration was under the CK (0.48 μg g^−1^). The minimum MBN value was under the PM + U treatment and the highest was in WS + U treatment.

The different effects of OAs plus urea in soil on DON content are shown in Fig. 1d. The maximum DON concentration was observed at the initial stage of incubation. In addition, compared with CK, OAs plus urea enhanced the maximum DON concentrations in the soils. The minimum values of DON content were observed on day 3, which were between 39.68 and 46.91 μg g^−1^ in OA plus urea treatments and 4.97 μg g^−1^ in CK. Then, the DON content gradually increased until the end of incubation. The final DON content was 7.14 μg g^−1^ in CK, while it was 60.53 μg g^−1^, 54.22 μg g^−1^, 54.79 μg g^−1^, and 48.14 μg g^−1^ in the PM + U, WS + U, CP + U, and IC + U groups, respectively. Additionally, all OA plus urea treatments, especially the PM + U treatment, enhanced the DON content in comparison to that in the CK over the whole incubation period.

### Abundance of AOA and AOB

**Figure 2:**
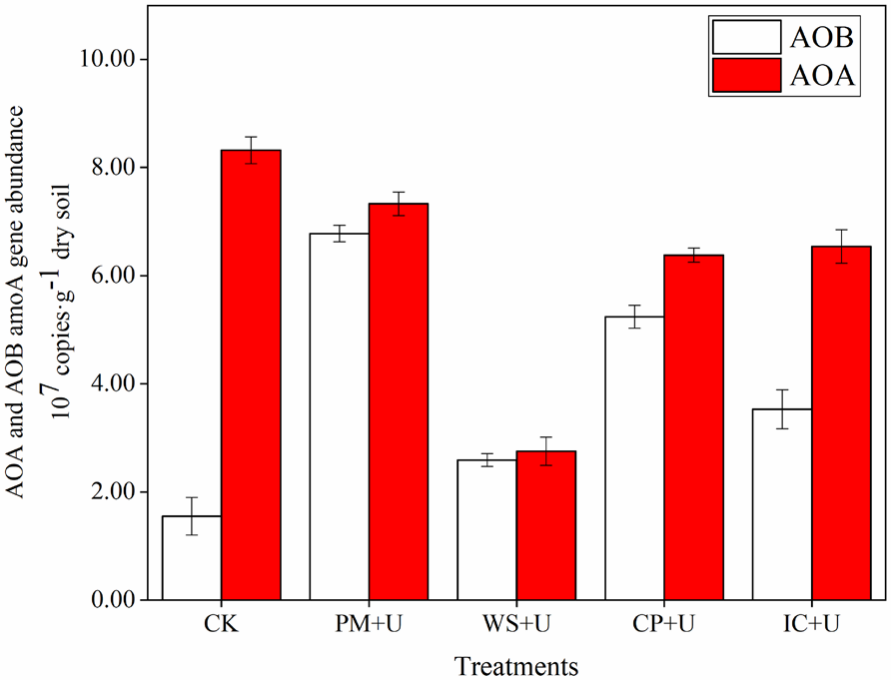
Abundance of archaeal (AOA) and bacterial (AOB) amoA genes among all treatments after the incubation (*AOA* ammonia-oxidizing archaea, *AOB* ammonia-oxidizing bacteria, *PM + U* pig manure plus urea, *WS + U* wheat straw plus urea, *CP + U* compost plus urea, *IC + U* improved compost plus urea).

The abundances of AOA and AOB are described based on their copy numbers, which ranged from 2.75 × 10^7^ to 8.32 × 10^7^ copies·g^−1^ dry soil and 1.55 × 10^7^ to 6.78 × 10^7^ copies g^−1^ dry soil, respectively, in the different treatments (Fig. 2). All OA plus urea treatments resulted in significant changes in the abundance of AOA and AOB. Compared with CK, the copy number of AOB was enhanced significantly in PM + U, WS + U, CP + U, and IC + U added soils by 4.37-, 1.67-, 3.38-, and 2.28- fold (*P* < 0.01). However, the copy number of AOA decreased by 11.90%, 66.95%, 23.32%, and 21.39% (*P* < 0.01), respectively.

Additionally, the abundance of AOA was significantly correlated with DON (*r* = − 0.946, *P* < 0.05) and MBN (*r* = − 0.940, *P* < 0.05), and the AOB abundance was significantly related to NH_4_^+^-N (*r* = 0.942, *P* < 0.05) and TN concentration (*r* = 0.991, *P* < 0.01).

### Diversity index of AOA and AOB

**Figure 3:**
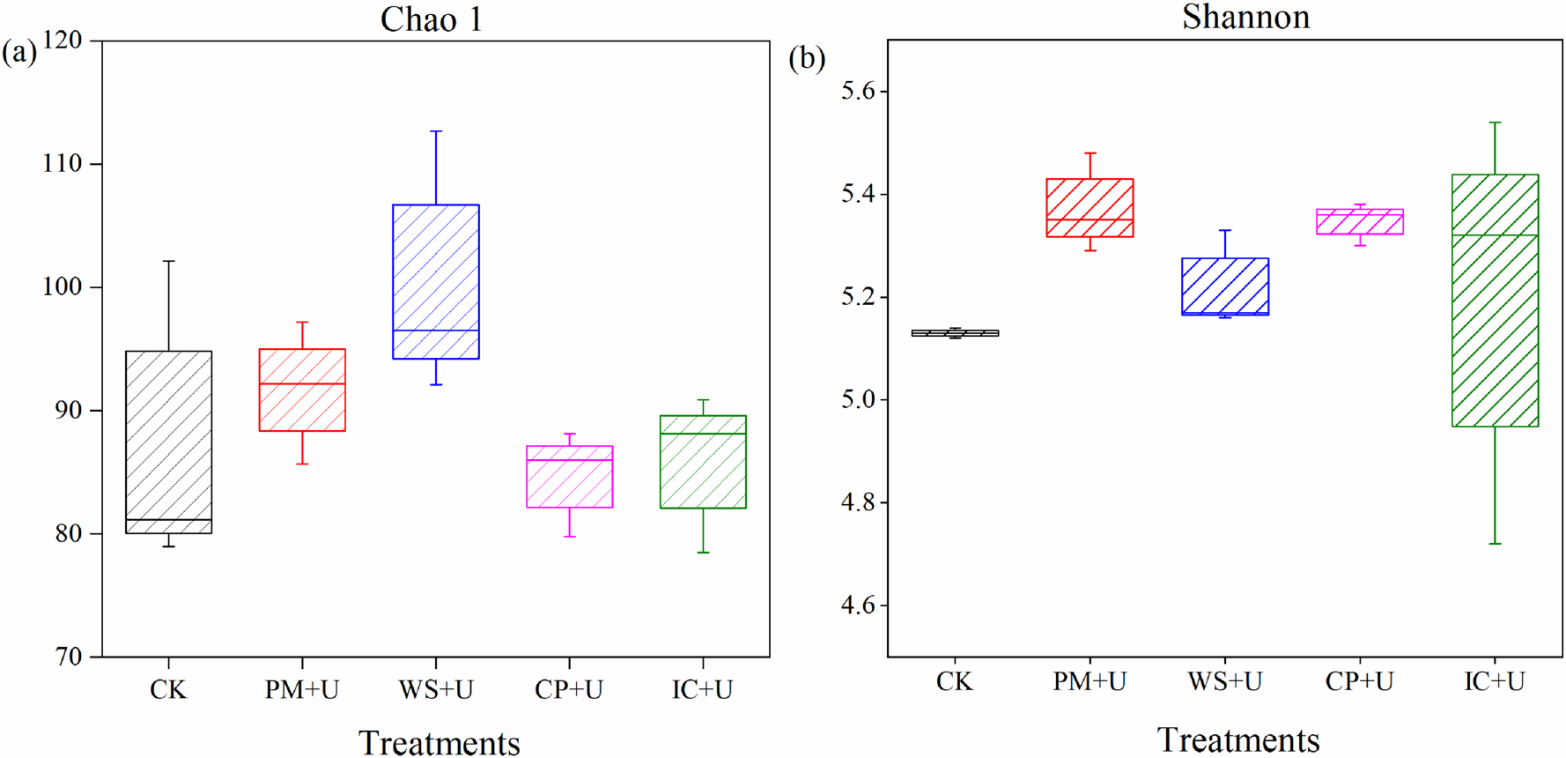
The community diversities of AOA (**a**) and AOB (**b**) among all treatments during the incubation (*AOA* ammonia-oxidizing archaea, *AOB* ammonia-oxidizing bacteria, *PM + U* pig manure plus urea, *WS + U* wheat straw plus urea, *CP + U* compost plus urea, *IC + U* improved compost plus urea).

Functional genes of AOA and AOB were quantitatively analyzed. A total of 41,165 and 24,910 high-quality AOA and AOB sequences, respectively, were obtained from 15 samples. In addition, 74–109 OTUs and 251–301 OTUs were assigned to the AOA and AOB sequences, respectively, at a 100% similarity level. The richness of AOA and AOB related genes is shown in Fig. 3. The OA plus urea treatments increased the richness of AOA by 0.81–14.87% compared with that in the CK (Chao 1). Furthermore, compared with that by the CP + U, IC + U, and CK groups, the PM + U and WS + U treatments enhanced the richness of AOA. Moreover, the richness of AOA in the WS + U treatment increased by 9.54% compared with that in the PM + U treatment. Additionally, the OAs plus urea increased the richness of AOB by 1.23–4.74% compared with the CK (Shannon index). However, there were no significant differences among the treatments. Compared with the IC + U (5.19) and WS + U (5.22) treatments, the richness of AOB was enhanced under the PM + U (5.37) and CP + U (5.35) treatments.

### Composition of AOA and AOB communities

**Figure 4:**
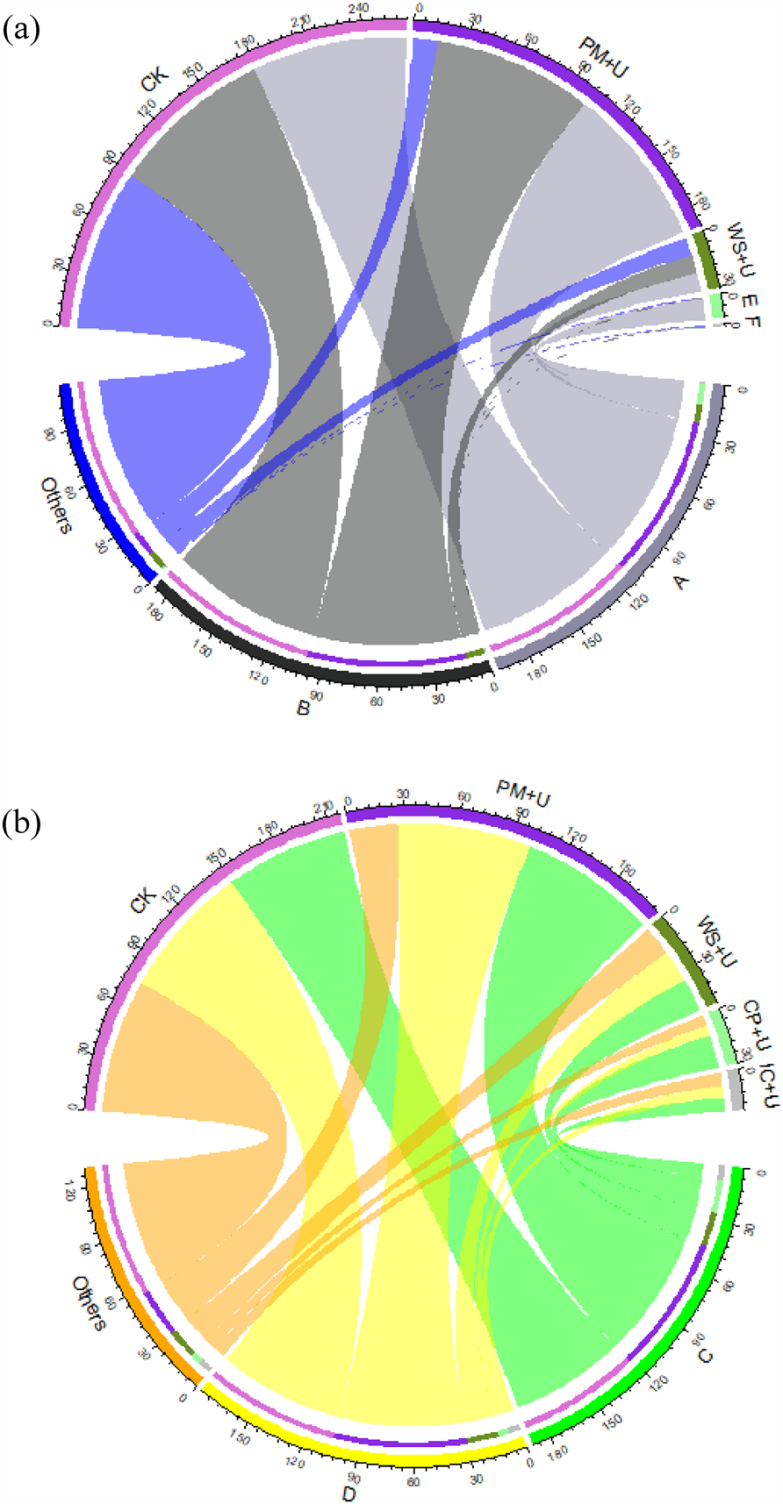
The community compositions of AOA (**a**) and AOB (**b**) among all treatments during the incubation (*AOA* ammonia-oxidizing archaea, *AOB* ammonia-oxidizing bacteria, A: *Unidentified_Thaumarchaeota*; B: *Unidentified_Crenarchaeota*; C: *Unclassified_Nitrosomonadales*; D: *Unclassified_Bacteria*, *PM + U* pig manure plus urea, *WS + U* wheat straw plus urea, *CP + U* (E): compost plus urea, *IC + U* (F): improved compost plus urea).

The predominant genes for the AOA-type ammonia oxidizer are shown in Fig. 4a. *Unclassified_Thaumarchaeota* was the most abundant gene in soil, and its relative abundance accounted for 85.86–89.46%. Except for the IC + U treatment, other OAs plus urea significantly increased the abundance of *Unclassified_Thaumarchaeota* compared with that in the CK (*P* < 0.05). However, the difference between OA plus urea treatments was not significant (*P* > 0.05). *Unclassified_Crenarchaeota* was the next most abundant gene in soil, accounting for 10.06–13.73%. All OAs plus urea significantly decreased the abundance of *Unclassified_Crenarchaeota* relative to that in the CK (*P* < 0.05); however, no significant difference was observed among the OA plus urea treatments.

As shown in Fig. 4b, *Proteobacteria* was the dominant phylum for AOB-type nitrifying genes in soil. *Unclassified_Nitrosomonadales* was the predominant gene in all treatments, accounting for 68.11–76.66%. The OAs plus urea significantly increased the abundance of *Unclassified_Nitrosomonadales* by 8.90–12.56% relative to that in the CK (*P* < 0.05). Among all OA plus urea treatments, the relative abundance of *Unclassified_Nitrosomonadales* increased under the CP + U and WS + U groups compared with that under the IC + U and PM + U groups (*P* < 0.05). *Unclassified_bacteria* was more abundant in soil, with a relative abundance of 16.78–26.18%. The OAs plus urea significantly decreased the abundance of *Unclassified_bacteria* relative to that in the CK (*P* < 0.05). Moreover, PM plus urea significantly enhanced the abundance of *Unclassified_bacteria* compared to that by other OA plus urea treatments.

### Correlation between soil properties and compositions of ammonia oxidizers

**Figure 5:**
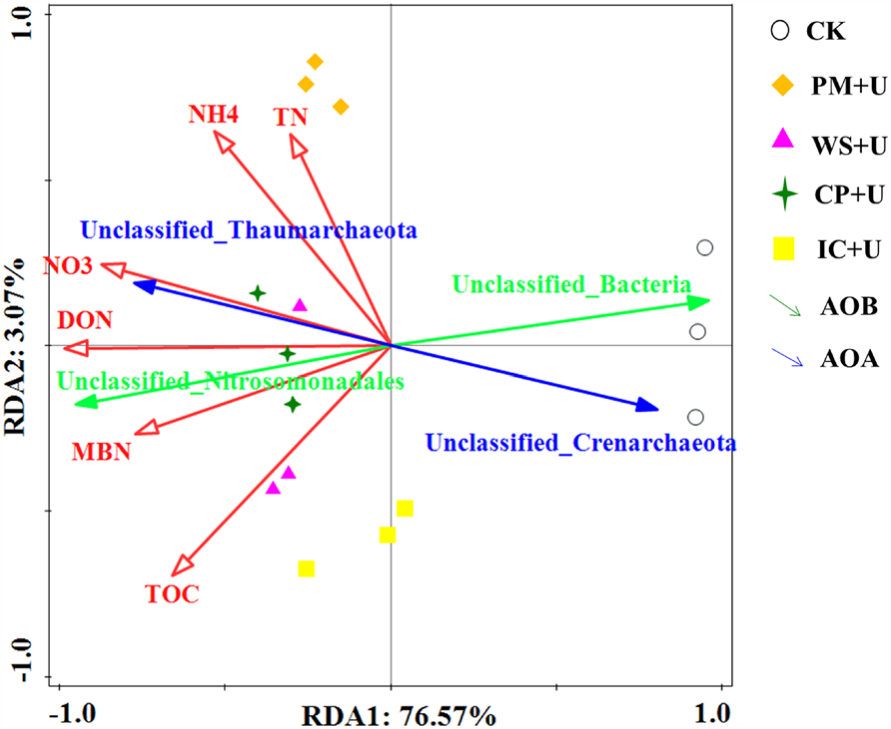
Redundancy analysis (RDA) of the relationship between soil properties and community structure of AOA and AOB among all treatments (*AOA* ammonia-oxidizing archaea, *AOB* ammonia-oxidizing bacteria, *PM + U* pig manure plus urea, *WS + U* wheat straw plus urea, *CP + U* compost plus urea, *IC + U* improved compost plus urea).

The correlation of various soil chemical properties and microbial communities was evaluated using RDA to explore their contribution to nitrification in OA plus urea treatments (Fig. 5). The soil properties explained 79.64% of the variations in bacterial communities. The most influential indicator was DON (74.1%), followed by NO_3_^−^-N (58.4%), MBN (45.9%), TOC (34.7%), NH_4_^+^-N (23.0%), and TN (8.3%). Therefore, these properties significantly influenced the variations in bacterial communities (AOA-type and AOB-type nitrifying genes) during the incubation. *Unclassified_Nitrosomonadales* belonging to the AOB *amo*A nitrification gene had a significantly positive association with DON (*r* = 0.934, *P* < 0.05) and NO_3_^−^-N (*r* = 0.920, *P* < 0.05). *Unclassified_bacteria* for AOB-type nitrifying genes were negatively associated with DON (*r* = − 0.973, *P* < 0.01). *Unclassified_Thaumarchaeota* (*r* = 0.940, *P* < 0.05) *and Unclassified_Crenarchaeota* (*r* = − 0.946, *P* < 0.05) belonging to AOA amoA nitrification genes were significantly positively related to DON.

## Discussion

### Nitrogen mineralization and nitrification

The release of available nitrogen, which can be absorbed by plants and microbes, is significant in the ecosystem. The principal nitrogen source for crops and microbes is from soil organic matter via mineralization and from urea via hydrolysis. The results showed that compared with the CK, the DON, MBN, MN, and net mineralized N content increased with the addition of OAs and urea. Under the same amount of urea application, the difference of mineralized nitrogen in OA plus urea treatments was attributed to OAs. This is because carbon sources can be rapidly utilized by microorganisms to enhance their activity and promote the release of nitrogen when OAs are added to the soil^25,26^.

The DON and NH_4_^+^-N content decreased, and that of MBN and NO_3_^−^-N gradually increased in the initial period of incubation. Microorganisms mineralize organic nitrogen into inorganic nitrogen using carbon sources and DON with the addition of OAs in OA plus urea treatments^27^. Meanwhile, inorganic nitrogen is immobilized by microorganisms and converted partly into MBN to enhance microbial activity. In this study, the highest net N mineralization was found in the PM + U group among all OA plus urea treatments during the 77-day incubation experiment, which may be owing to the lower C/N ratio of PM than that in the other three kinds of OAs^28^. Whitehead et al. indicated that organic manure with C/N lower than 20 could release more mineralized N by mineralization, which was consistent with this finding^29^. The C/N proportion of CP was slightly higher than that of PM, but the total MN in CP + U and IC + U treatments was significantly lower than that of the PM + U treatment. This is attributed to the large ratio of complex organic nitrogen and stable materials^30^. The finding was consistent with the results obtained by Hartz et al. and Zaman et al.^31,32^. However, OAs with higher C/N can immobilize more N^17^. In this study, the lowest MN released was under the WS + U soil, which was because its C/N was significantly higher than that of other OAs. This can be confirmed by the highest MBN in the WS + U amended soil. Furthermore, other studies reported that N mineralization in soil amended with clover residues was varied between 30 and 60%^33,34^. Hartz et al. also found that N mineralization in the soil containing manure and compost was 7% and 1%, respectively^31^. In this study, the proportion of net N mineralization to total N applied in the WS + U, PM + U, CP + U and IC + U group was 19.46%, 33.96%, 26.56% and 25.68% after the incubation, respectively. There were three explanations for the differences: one was the duration of the incubation experiment (77d), which was shorter than other experiments; the other was the temperature set in this study. The NH_3_ emission was promoted in the aerobic environment with an appropriate temperature (25 °C), thus reducing the amount of nitrogen mineralization in the soil; the third was the characteristics and composition of the organic materials.

In addition, the addition of OAs plus urea is conducive to the activities of nitrifying bacteria. The NH_4_^+^-N produced from the mineralization of organic nitrogen and the hydrolysis of urea was converted into NO_3_^−^-N with the addition of OAs plus urea, which gradually reduced the content of NH_4_^+^-N in soil. The NO_3_^−^-N content was largely increased in the PM + U treatment. There are two possible explanations: one, PM amendment can produce more NH_4_^+^-N in soil by promoting the mineralization of organic nitrogen, and thus providing sufficient substrate for nitrification and two, the mineralization of organic nitrogen was greater than the fixation of mineral nitrogen with lower C/N in PM^35^. The NO_3_^−^-N content tended to be stable, indicating that decomposable organic matter gradually decreased and energy providing material gradually depleted with nitrification and fixation. In contrast, the lowest NO_3_^−^-N content was observed under the WS + U group. On the one hand, the lower NH_4_^+^-N does not provide sufficient substrate for nitrification. On the other hand, the fixation of NO_3_^−^-N produced was greater than mineralization with the highest C/N in WS^19,36^.

### Community abundance of AOA and AOB

Nitrifying bacteria are active in soil biological nitrification^1^. The results of this study showed that the addition of OAs and urea promoted the activity of nitrifying bacteria in soil. Similarly, in this study, we do not consider the effect of various organic matter on urea hydrolysis, but only think that the difference of abundance in nitrifying bacteria in different OA plus urea treatments was caused by different types of OAs. Many studies have shown that organic fertilization can increase the number of nitrifiers in soil^37,38^, which was owing to the fertilizer that provided the substrate and energy for nitrifying bacteria and promoted their growth and reproduction^39,40^. Furthermore, some intermediate metabolites may be produced during the mineralization of organic nitrogen, which may be beneficial to the growth of AOB and stimulate soil nitrification^41^.

The copy numbers of AOA were larger than those of AOB during the incubation period in this study, which was consistent with that reported in other studies in most soils^42,43^. The application of different OAs and urea significantly enhanced the copy number of AOB, but decreased that of AOA. This finding is consistent with the finding reported by Taylor et al., who confirmed that fertilizer application had a larger effect on AOB than on AOA^8^. Furthermore, it was also confirmed that fertilization could significantly stimulate AOB, but had no impact on the AOA community in paddy soil^44^. However, the result was in contrast with that reported by Wessen et al.^45^, who found that the application of organic carbon increased AOA abundance in a long-term experiment. The growth of AOA is favored with high NH_4_^+^ content, whereas AOB are well adapted to low NH_4_^+^ contents in the environment^46^. In this study, NH_4_^+^-N was significantly related to AOB abundance (*r* = 0.924, *P* < 0.05), but not to that of AOA. Therefore, the higher NH_4_^+^-N content in OAs could explain the higher AOB and lower AOA abundance in the soil.

The increased abundance of the amoA gene may have contributed to the increase in soil nitrification. In this study, AOB abundance was positively correlated with NO_3_^−^-N (*r* = 0.835, *P* > 0.05), whereas AOA had a negative correlation with NO_3_^−^-N (*r* = − 0.663, *P* > 0.05). It can be concluded that AOB was more functional for nitrification. Jia and Conrad also confirmed that AOB contributed more in terms of function^14^. However, Leininger et al. have shown that nitrification is largely driven by AOA in ecosystems^47^. These findings illustrated that although the AOA abundance was larger than that of AOB, AOB contributed more to nitrification than AOA in soils. Di et al. also confirmed this conclusion. In addition, the highest AOB abundance could explain the strong nitrification in the PM + U group^48^. However, the lowest abundance of AOB and weakened nitrification was under the WS + U treatment among all OA plus urea amended soils.

### Community structure of AOA and AOB

In this study, OAs plus urea increased the diversity index of AOA and AOB in soil relative to that in the CK, demonstrating that OAs plus urea could promote the growth of ammonia oxidizers during incubation. In addition, OAs plus urea increased the community diversity of AOB considerably compared to that of AOA. The highest AOB community diversity was observed in the PM + U treatment, which indicated that PM plus urea could promote the growth of AOB more than that by other OAs plus urea because it was easier to decompose and mineralize than the other OAs. The result was different from that reported by Schauss et al., who found that manure amendment favored the growth of AOA^49^. The PCA results reflected that the compositions of AOA and AOB communities varied in response to OAs. Thus, different organic carbon levels significantly influenced the structure of AOA and AOB during the incubation process. However, the application of OAs and urea affected the structure of the AOA community slightly, but clearly influenced the structure of the AOB community. The main reasons could be ascribed to various responses of AOA and AOB to OAs. The results demonstrated that the AOB community structure was more sensitive than that of AOA to various OAs plus urea. This finding was consistent with the research reported by Wu et al.^50^ that the amendments of urea and rice straw varied the AOB community structure, but not that of the AOA in a 22-year long-term experiment.

AOA and AOB contribute differently to soil nitrification depending on environmental and management measures. In this study, the application of OAs plus urea significantly changed soil properties at the end of the incubation period, and the changes in soil properties affected the changes in soil ammonia-oxidizing microorganisms. Soil NO_3_^−^-N and DON were important factors affecting the distribution of ammonia-oxidizing microorganisms. *Unclassified_Nitrosomonadales* belonging to AOB amoA ammonia-oxidizing genes had a significantly positive relationship with NO_3_^−^-N (*P* < 0.05). The AOB was found to be responsible for soil nitrification. This finding was consistent with that of Jia and Conrad^14^, who found that AOB played an important role in nitrification in some agricultural soils under different fertilization practices. However, some studies indicated that AOA, but not AOB, played a dominant role in nitrification in acidic soil^14,43,51^. Therefore, further research is needed to confirm the contribution of AOA and AOB to nitrification under various fertilization practices. In addition, *Unclassified_bacteria* for the AOB-type ammonia-oxidizing gene was significantly associated with DON (*P* < 0.01) compared with the other dominant genera. DON is mainly produced by the mineralization of organic nitrogen. The results indicated that AOB contributed more to the mineralization of organic nitrogen than did AOA with different OAs plus urea.

## Conclusions

The results showed that OAs plus urea promoted mineralization and nitrification in soil. The NH_4_^+^-N mineralized by organic nitrogen from OAs enhanced AOB abundance, but reduced the AOA abundance. AOA outnumbered AOB, whereas AOB contributed more to nitrification than AOA with the different OAs plus urea in soil. Soil NO_3_^−^-N and DON were important influencing factors for the structure of AOA and AOB communities. The result illustrated that the pig manure plus urea application outperforms wheat straw plus urea, compost product plus urea, and improved compost plus urea, which is an effective practice to release more available nitrogen in agricultural systems.

## Data Availability

All data generated or analysed during this study are included in this published article.
